# Resensitizing Paclitaxel-Resistant Ovarian Cancer via Targeting Lipid Metabolism Key Enzymes CPT1A, SCD and FASN

**DOI:** 10.3390/ijms242216503

**Published:** 2023-11-19

**Authors:** Qinsiyu Ma, Zhan’ao Liu, Tengyu Wang, Pengfei Zhao, Mingrui Liu, Yifang Wang, Weitong Zhao, Ying Yuan, Shuo Li

**Affiliations:** Department of Biochemistry & Molecular Biology, School of Life Sciences, China Medical University, Shenyang 110122, China; mqsy20020529@outlook.com (Q.M.); liuzhanao1@outlook.com (Z.L.); 2021352609@cmu.edu.cn (T.W.); zhaopengfei10@outlook.com (P.Z.); baichamr@outlook.com (M.L.); wwblbhsgxh@outlook.com (Y.W.); zhaoweitongwhitley@outlook.com (W.Z.)

**Keywords:** epithelial ovarian cancer, paclitaxel resistance, lipid metabolism, *CPT1A*, *FASN*, *SCD*

## Abstract

Epithelial ovarian cancer (EOC) is a lethal gynecological cancer, of which paclitaxel resistance is the major factor limiting treatment outcomes, and identification of paclitaxel resistance-related genes is arduous. We obtained transcriptomic data from seven paclitaxel-resistant ovarian cancer cell lines and corresponding sensitive cell lines. Define genes significantly up-regulated in at least three resistant cell lines, meanwhile they did not down-regulate in the other resistant cell lines as candidate genes. Candidate genes were then ranked according to the frequencies of significant up-regulation in resistant cell lines, defining genes with the highest rankings as paclitaxel resistance-related genes (PRGs). Patients were grouped based on the median expression of PRGs. The lipid metabolism-related gene set and the oncological gene set were established and took intersections with genes co-upregulated with PRGs, obtaining 229 co-upregulated genes associated with lipid metabolism and tumorigenesis. The PPI network obtained 19 highly confidential synergistic targets (interaction score > 0.7) that directly associated with CPT1A. Finally, FASN and SCD were up-stream substrate provider and competitor of CPT1A, respectively. Western blot and qRT-PCR results confirmed the over-expression of *CPT1A*, *SCD* and *FASN* in the A2780/PTX cell line. The inhibition of *CPT1A*, *SCD* and *FASN* down-regulated cell viability and migration, pharmacological blockade of CPT1A and SCD increased apoptosis rate and paclitaxel sensitivity of A2780/PTX. In summary, our novel bioinformatic methods can overcome difficulties in drug resistance evaluation, providing promising therapeutical strategies for paclitaxel-resistant EOC via taregting lipid metabolism-related enzymes.

## 1. Introduction

Epithelial ovarian cancer (EOC) is one of the most lethal gynecological malignant tumors. According to the global cancer statistics 2022, EOC is the fifth most common cancer among women worldwide [1]. Currently, the standard treatment for EOC consists of cytoreductive surgery and chemotherapy combining platinum compounds (including cisplatin and carboplatin) and taxanes (including paclitaxel and docetaxel). Paclitaxel promotes the assembly of microtubule polymers composed of α/β-tubulin heterodimers by binding tightly to β-tubulin, stabilizing microtubules, and accumulating disorganized microtubule filaments [2,3,4,5]. In this way, paclitaxel causes mitosis failure, leading to cell cycle arrest at the G2/M phase, thus inhibiting cancer cell proliferation. Acquired paclitaxel resistance is the major cause for failed treatments, and it leads to poor prognosis [6]. Mostly the mechanisms of acquired paclitaxel resistance include alterations of microtubules and mutations of apoptosis-related genes that result in cell death resistance. The over-expression of the ATP-binding cassette (ABC) family, which plays the role of a multidrug resistance (MDR) transporter, increases cellular drug efflux and causes multiple drug-resistant phenotypes [7,8,9,10]. Exploring the fundamental mechanisms of acquired paclitaxel resistance will provide potential targets and novel therapeutic perspectives for clinical treatments of ovarian cancer.

It is widely accepted that metabolic alterations have the potential to contribute to therapy resistance. The changes in metabolic pathways indicate not simply nutrient deficiency, but also a stress response that leads to chemotherapy resistance occurrence. Increased reliance on mitochondrial energy metabolism, in particular oxidative phosphorylation (OXPHOS), has been reported as a distinctive hallmark of chemotherapy-resistant cancer cells in several tumor types [11,12]. The shift towards OXPHOS may confer to cancer cells some metabolic/growth advantages that allow them resistance to chemotherapy. Recently, it has been proposed that chemotherapy-resistant cells exhibit increased fatty acid (FA) uptake, accompanied by decreased glucose uptake and lipogenesis, which indicates reprogramming from glucose to FA-dependent anabolic and energy metabolism. The rapid proliferation of cancer cells demands an adequate energy supply, which results in over-activation of fatty acid de novo synthesis, causing lipid droplet accumulation and also promoting the rate of β-oxidation to support the rapid division of cancer cells [13]. Drug-resistant cells exhibit a spcial membrane lipid composition of intracellular organelles including mitochondria and endoplasmic reticulum (ER) containing a lower amount of oxidizable fatty acids; hence, they are more resistant to oxidative stress and chemotherapy-induced apoptosis. Phospholipids are rich in very long saturated fatty acid chains, and a relative increase in cholesterol affects the expression, recycling and biological activities of the proteins concentrated in these domains, including ABC transporters, hence favoring drug binding and release [14,15]. Therefore, studying lipid metabolism reprogramming may uncover new clinical opportunities for paclitaxel-resistant ovarian cancer patients [16].

In previous research, we noticed that lipid metabolism-related enzyme carnitine palmitoyltransferase 1A (CPT1A) differentially expressed with significance in paclitaxel-resistant EOC cell lines. CPT1A is located on the inner membrane of mitochondria, which is the rate-limiting enzyme of fatty acid oxidation (FAO) that catalyzes the transfer of the long-chain acyl group in the acyl-CoA ester to carnitine, allowing fatty acids entrance to the mitochondrial matrix for oxidation [17,18]. Recent research has pointed to the crucial role of CPT1A mediating FAO as an essential source of NADH, FADH${}_{2}$ and ATP, providing survival advantage to cancer, as well as being the carbon source for the synthesis of nucleoside metabolic [19,20,21,22]. Meanwhile, CPT1A also shares multiple connections with many other cellular signaling pathways, including c-MYC or AMPK in breast cancer, and promotes cancer proliferation, metastasis or therapeutic resistance through several oncogenic signaling pathways, such as PI3K/AKT/mTOR, VEGF, ERK and Src pathways [23,24,25,26,27,28]. Over-expression of CPT1A correlates strongly with poor patient outcomes of ovarian cancer. Huang et al. [29] identify CPT1A as the biomarker of platinum resistance through multiomic analysis. Therefore, CPT1A is a desirable target for the treatment of paclitaxel resistance in ovarian cancer.

Furthermore, we focused on selecting lipid metabolism-related enzymes as candidate targets for resensitizing paclitaxel resistance in ovarian cancer. We further discovered stearoyl CoA desaturase (SCD) and fatty acid synthase (FASN) due to their roles as key enzymes in lipid metabolic pathways. SCD is an endoplasmic reticulum enzyme, and it is the main source of Δ${}^{9}$-monounsaturated fatty acid (MUFA) generation. MUFAs function as major components of cell membrane phospholipids and cholesterol esters. Previous research demonstrated that SCD is a biochemical hallmark of cancer cells and that it modulates fatty acid composition in cancer. *SCD* is over-expressed in multiple malignant tumors, which plays a vital role in regulating proliferation, signal transduction, metastasis and modulates lipid metabolism via reducing fatty acid oxidation to foster lipogenesis [30]. SCD also supports tumorigenesis through stimulating the Wnt pathway, as well as the PI3K/AKT/HIF2, and NF-κB pathways [31,32]. FASN is widely known as the central enzyme of de novo fatty acid synthesis. In cancer cell lines, *FASN* over-expression was found to cause chemotherapy resistance induced by drug-containing media, suggesting that FASN may be a causing factor of cancer chemoresistance. This phenotype is considered to associate with poor prognosis and a higher risk of cancer recurrence [33,34,35]. This study explored the expression of hub genes in acquired paclitaxel resistance cell lines based on lipid metabolism reprogramming processes, which are capable of evaluating the potential targets causing failure of paclitaxel delivery in EOC treatments and providing new clinical therapeutical strategies.

## 2. Results

### 2.1. Screening, Validation of PRGs and Univariate Regression Analysis

**Figure 1:**
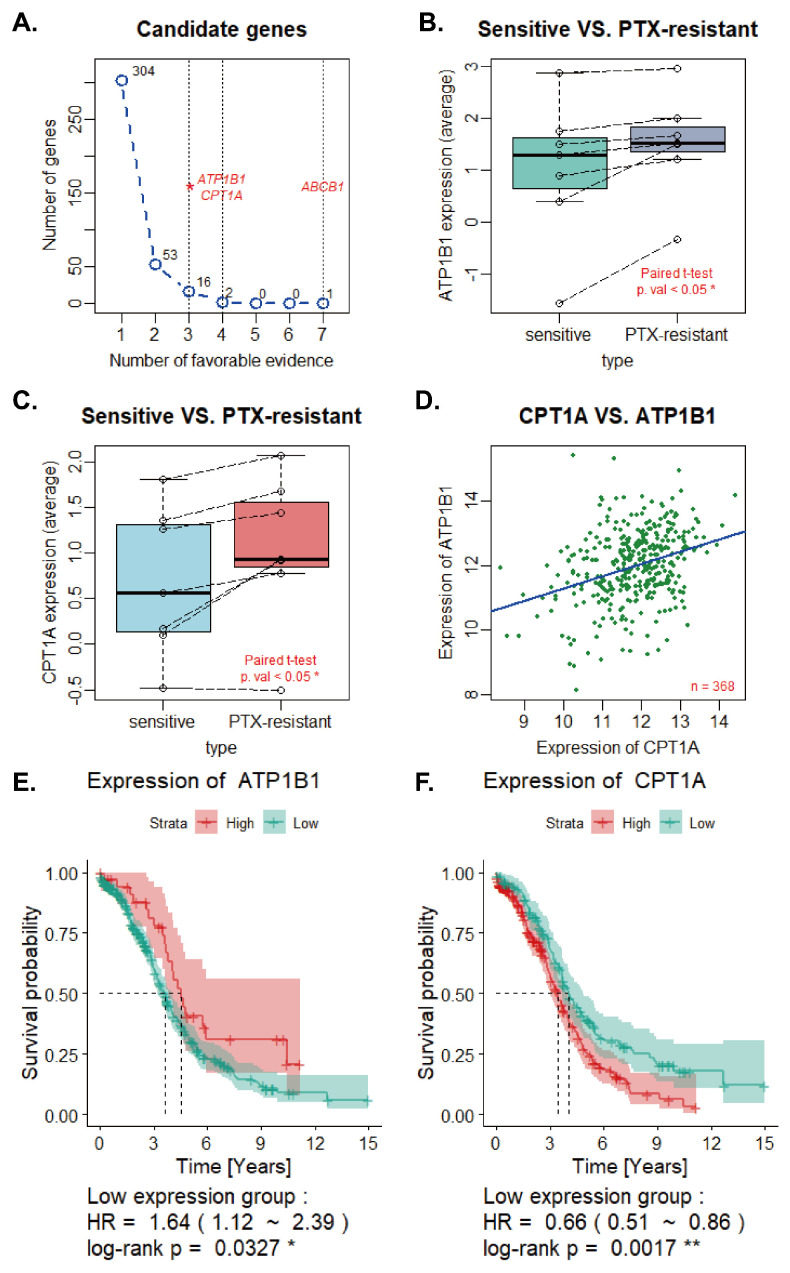
(**A**) Number of favourable evidences of candidate genes. (**B**,**C**) Expression levels of *ATP1B1* and *CPT1A* in sensitive cell lines and resistant cell lines. (**D**) Expression levels of *CPT1A* and *ATP1B1* in pathological samples. (**E**,**F**) Overall survival analysis of ovarian cancer patients with lower or higher expression levels of *CPT1A* and *ATP1B1* were estimated using the Kaplan–Meier method by log-rank test according to data from the GDC database. * represents *p* < 0.05; ** represents *p* < 0.01.

We compared expression levels of genes from paclitaxel-resistant cell lines and their original sensitive cell lines. In total, 19 candidate genes (*ACSS1*, *AK5*, *ALCAM*, *CHRD*, *CLDN1*, *CLDN16*, *GALNT10*, *HERC5*, *LAMB1*, *NFE2L3*, *OSTF1*, *PGM2*, *PODXL*, *PRKD3*, *SLC4A8*, *THBS1*, *CPT1A*, *ATP1B1*, *ABCB1*) were confirmed according to our previously mentioned criteria (Figure 1A); among them, *CPT1A*, *ATP1B1*, *ABCB1* were considered as PRGs in ovarian cancer. *ABCB1* showed prominent high expression in all seven paclitaxel-resistant cell lines, and *CPT1A* and *ATP1B1* were the up-regulated candidate genes in four resistant cell lines. The expression levels of other 16 candidate genes were significantly elevated in three resistant cell lines.

The expression levels of *CPT1A* and *ATP1B1* were remarkably elevated in seven paclitaxel-resistant cell lines compared to sensitive cell lines (Figure 1B,C) (CPT1A: *p*.val = 0.01114 *; ATP1B1: *p*.val = 0.01729 *). Furthermore, we developed a univariate regression model using *CPT1A* expression in pathological tissues as a predictor of *ATP1B1* expression (Figure 1D), and we obtained the following formula: ATP1B1= 0.37822×CPT1A + 7.50708 (R${}^{2}$ = 0.104, *p*.val = 2.377 $\times \;10^{-10}$ ****).

A positive correlation was discovered between *CPT1A* and *ATP1B1* expression in pathological tissues (Pearson correlation test: r = 0.3224573, *p*.val = 2.377 $\times \;10^{-10}$ ****).

### 2.2. Overall Survival Analysis and Identification of Synergistic Genes

**Figure 2:**
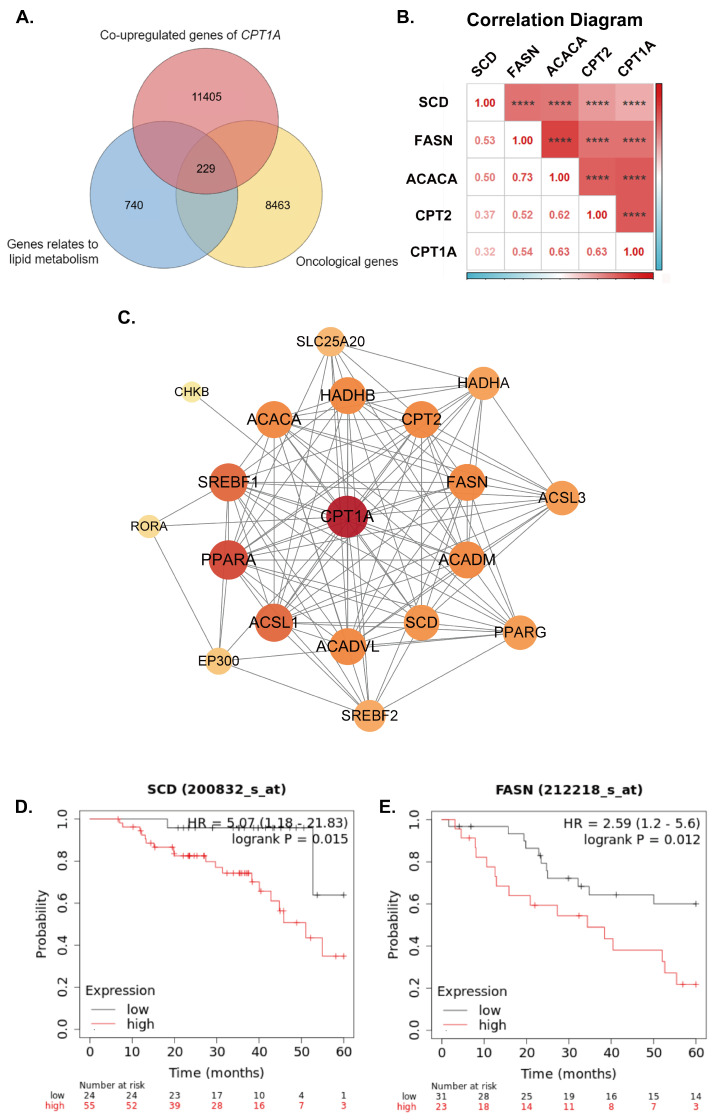
(**A**) Venn diagram for the screening of the core genes in three gene sets. (**B**) Correlation analysis among the expression levels of *SCD*, *FASN*, *ACACA*, *CPT2* and *CPT1A*. The significance expression levels between synergistic genes and CPT1A expressed by asterisks (****). (**C**) A total of 19 synergistic proteins having high confidential connections with CPT1A in the STRING database. (**D,E**) The OS analysis of ovarian cancer patients with low and high expression levels of *SCD* and *FASN* were estimated with the Kaplan–Meier method by log-rank test according to data from the TCGA database.

The selected patients and their disease characteristics for the histologic subtypes are shown in Table 1. Overall survival analysis was performed to investigate the prognostic effects of *CPT1A* and *ATP1B1* in ovarian cancer (Figure 1E,F). The results suggested *CPT1A* and *ATP1B1* were significantly related to survival time of EOC patients (CPT1A: *p*.val = 0.0017, ATP1B1: *p*.val = 0.0327). Therefore, high expression of *CPT1A* correlates with poor prognosis of patients, while a higher expression of *ATP1B1* correlates with a better prognosis. The previously established lipid metabolism-related gene set and the oncological gene set were applied to select genes co-upregulated with *CPT1A* (log2FC ≥ 0.6 and *p*.val ≤ 0.05) in the higher *CPT1A* expression group, obtaining 229 synergistic genes of *CPT1A* (Figure 2A).

In order to understand the underlying mechanisms of CPT1A in drug-resistant ovarian cancer, a PPI network of CPT1A and 229 synergistic genes was constructed using the STRING database. As shown in Figure 2C, 19 CPT1A interacting proteins were confirmed to have high confidential connections with CPT1A, including ACACA, ACADM, ACADVL, ACSL1, ACSL3, CHKB, CPT1A, RORA, EP300, SCD, HADHB, SREBF2, PPARG, FASN, HADHA, SREBF1, PPARA, SLC25A20, CPT2. Finally, intersection with drug resistance-associated genes was reported in the review of Yang et al. (*ACLY*, *ACACA*, *ACACB*, *FASN*, *CPT1A*, *CPT1B*, *CPT1C*, *CPT2*, *SCD*), confirming *ACACA*, *FASN*, *CPT2* and *SCD* as LRGs that function synergistically with *CPT1A* in the occurrence of paclitaxel resistance in ovarian cancer.

We further investigated the expression correlation between *CPT1A* and four sysnergistic genes (*ACACA*, *FASN*, *CPT2* and *SCD*) in pathological tissues (Figure 2B), indicating the expression levels of synergistic genes positively related to the expression level of *CPT1A* with significance. CPT1A, SCD and FASN may form an adjustable lipid metabolism enhancing pathway with CPT1A as the center, which plays a key synergistic role in the development of paclitaxel resistance in ovarian cancer. Overall survival analyses of *FASN* and *SCD* were performed, indicating that the over-expression of *FASN* and *SCD* in EOC patients leads to shorter survival time (Figure 2D,E).

### 2.3. Validation of Gene Expression Levels of *CPT1A*, *SCD* and *FASN* in Cultured Cell Lines

**Figure 3:**
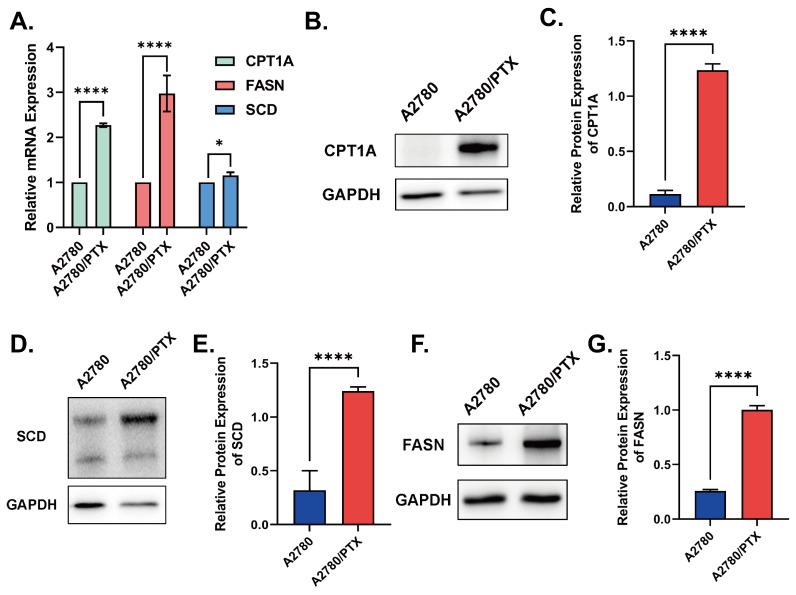
(**A**) qRT-PCR results of mRNA expression of *CPT1A*, *SCD* and *FASN* in A2780 and A2780/PTX cell lines; (**B**–**G**) Western blot results of protein expression of CPT1A, SCD and FASN in A2780 and A2780/PTX cell lines. All experiments were performed in triplicate. * represents *p* < 0.05; **** represents *p* < 0.0001.

We explored the expression levels of the three targets in A2780 and A2780/PTX ovarian cancer cell lines. qRT-PCR was performed to detect the mRNA expression of the three targets in OC cell lines. It was noticed that mRNA expressions of *CPT1A*, *SCD* and *FASN* were elevated in A2780/PTX compared to A2780 (Figure 3A); a higher protein expression of CPT1A, SCD and FASN in A2780/PTX was also indicated by Western blot anaylsis results (Figure 3B–G). We noticed the protein over-expression of SCD is of significance, yet SCD mRNA expression in the A2780/PTX cell line is marginally higher than that in A2780. We suggest that there are transcriptional and translational regulation or differences in mRNA and protein stability behind the phenomenon. The detailed mechanisms still require extensive exploration. The results indicated a higher mRNA and protein expression of CPT1A, SCD and FASN in the paclitaxel-resistant EOC cell line, which further validated the results obtained by bio-informatic methods.

### 2.4. Down-Regulation of *CPT1A*, *SCD* and *FASN* Resensitizes A2780/PTX to Paclitaxel and Inhibits the Capabilities in Cell Proliferation and Metastasis

**Figure 4:**
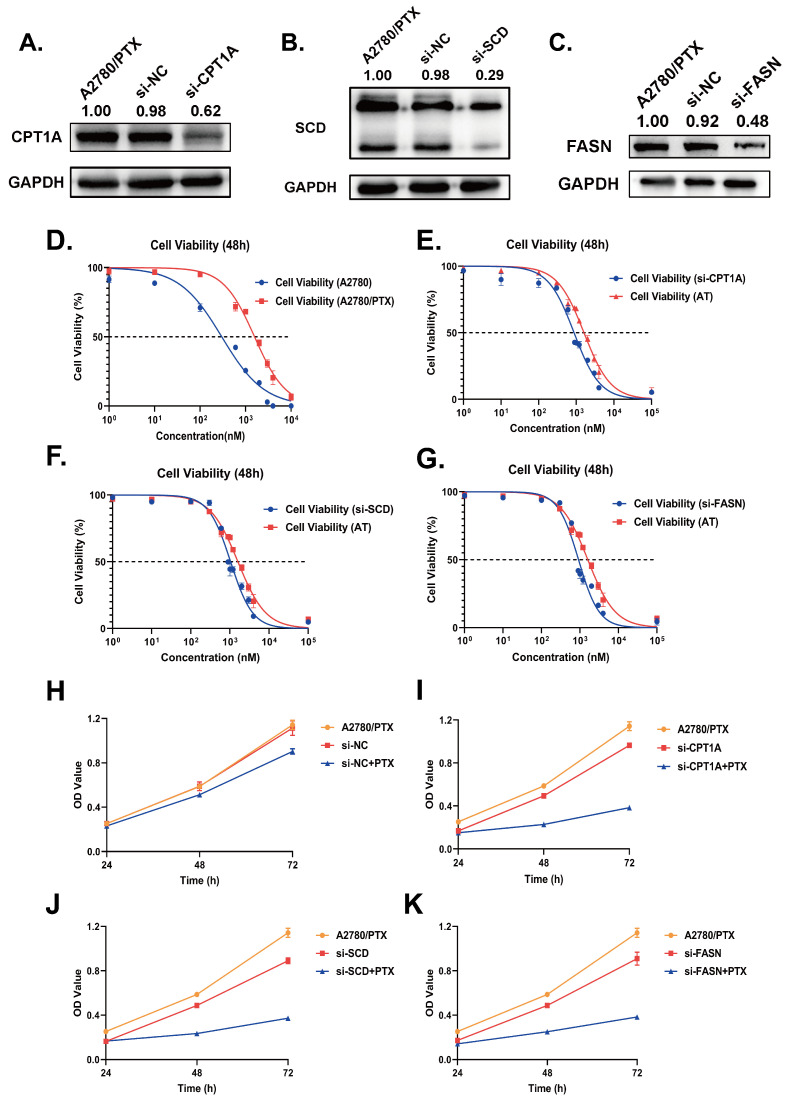
(**A**–**C**) Protein expression validation of CPT1A, SCD and FASN 48 h after siRNA knockdown; (**D**–**G**) IC50 curves calculated based on CCK-8 results 48 h after siRNA knockdown of *CPT1A*, *SCD* and *FASN* and treated with paclitaxel (0, 0.001, 0.01, 0.1, 0.3, 0.6, 0.9, 1.0, 1.2, 2.0, 3.0, 4.0, 100.0 mM); (**H**–**K**) OD values of applying siRNA with or without paclitaxel (1000 nM) at a 450 nm wavelength. All experiments were performed in triplicate.

To explore the functions of *CPT1A*, *SCD* and *FASN* in the A2780/PTX cell line, siRNA knockdown was performed to down-regulate the expression of *CPT1A*, *SCD* and *FASN*, As shown in Figure 4A–C, the protein expression levels were significantly decreased in A2780/PTX 48h after siRNA transfection, which indicated a satisfying knockdown efficiency.

The IC50 value of A2780/PTX is 1574 ± 89 nM, the IC50 value of A2780 is 299.7 ± 56.8 nM, and the resistance index (RI) is 5.2519. Compared to the control group (transfected using si-NC), after transfection by si-CPT1A, si-SCD and si-FASN, the IC50 values towards paclitaxel were reduced from 1589.5 ± 62.5 nM to 874.3 ± 53.1 nM, 942.1 ± 49.9 nM and 820.8 ± 53.7 nM (Figure 4D–G), respectively. We exhibited the OD values under different siRNA treatments (Figure 4H–K); inhibition at transcription level down-regulated the proliferation ability of A2780/PTX cells.

**Figure 5:**
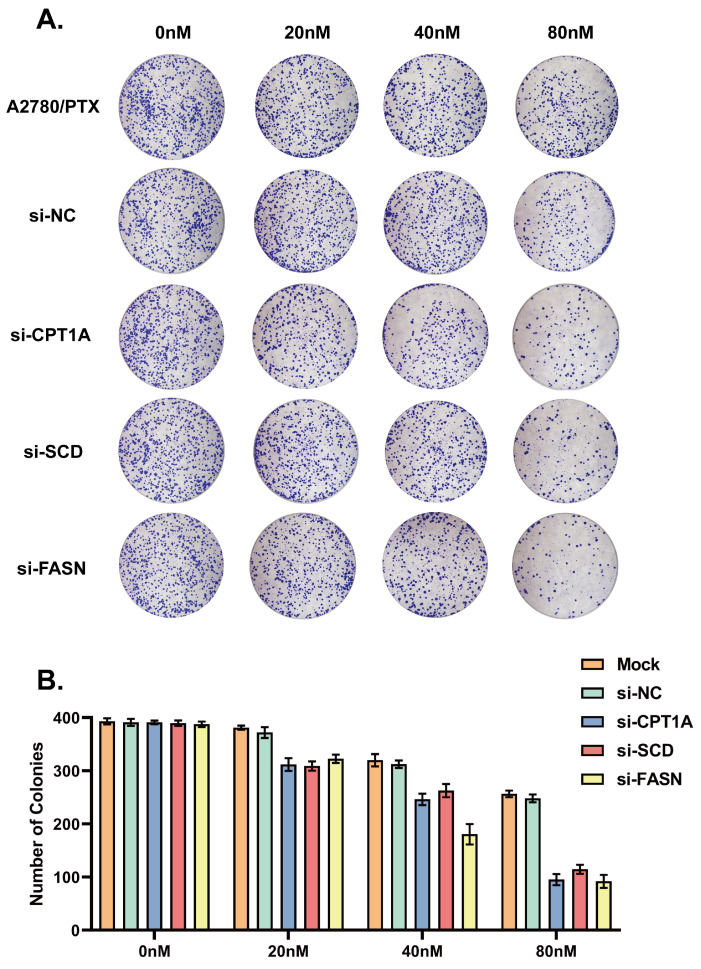
(**A**) Colony formation assay results 48 h after siRNA knockdown and treated with paclitaxel (0, 0.02, 0.04, 0.08 mM). (**B**) Graph showing the number of colonies under different concentrations of paclitaxel and different siRNA knockdown conditions. All experiments were performed in triplicate.

Colony formation assays were also performed. A2780/PTX cells transfected with si-CPT1A, si-SCD and si-FASN were treated with paclitaxel at concentrations of 20 nM, 40 nM and 80 nM for 14 days. Colonies formed by si-CPT1A-, si-SCD- and si-FASN-transfected A2780/PTX cells substantially decreased in a concentration-dependent manner compared to si-NC-transfected cells after paclitaxel treatment (Figure 5A,B).

**Figure 6:**
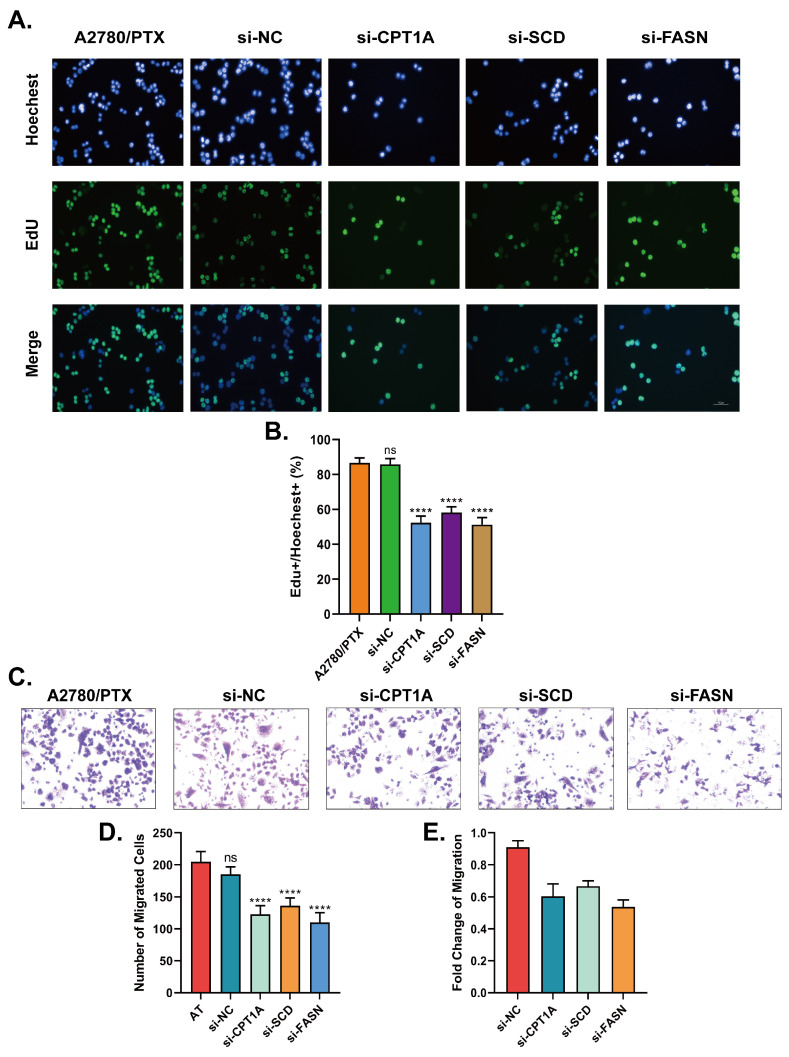
(**A**) EdU assay results of A2780/PTX- and siRNA-transfected cells 48 h after siRNA knockdown of *CPT1A*, *SCD* and *FASN*. (**B**) Graph showing EdU+/Hoechest+ ratios of different knockdown conditions with data presenting as mean ± standard deviation. (**C**) Results of Transwell migration assay indicated the capabilities of metastases of A2780/PTX cells 48 h after siRNA knockdown; (**D**,**E**) Graph showing the number and fold changes in migrated cells of different siRNA knockdown conditions. All data are presented as mean ± standard deviation. All experiments were performed in triplicate. **** represents *p* < 0.0001; ns represents not significant.

The EdU assay was further performed to confirm the proliferation suppression effect after transfection by si-CPT1A, si-SCD and si-FASN; the capability of proliferation obviously decreased according to EdU+/Hoechest+ ratios (Figure 6A,B). Transwell migration assay was performed to assess whether the inhibition of *CPT1A*, *SCD* and *FASN* can affect metastasis capabilities of A2780/PTX cells in the absence of paclitaxel. Migration decreased by approximately 30%, 25% and 35% when *CPT1A*, *SCD* and *FASN* were knocked down in A2780/PTX cells, respectively (Figure 6C–E).

### 2.5. Inhibitors of CPT1A, SCD and FASN Restore Sensitivity towards Paclitaxel and Induce Apoptosis

**Figure 7:**
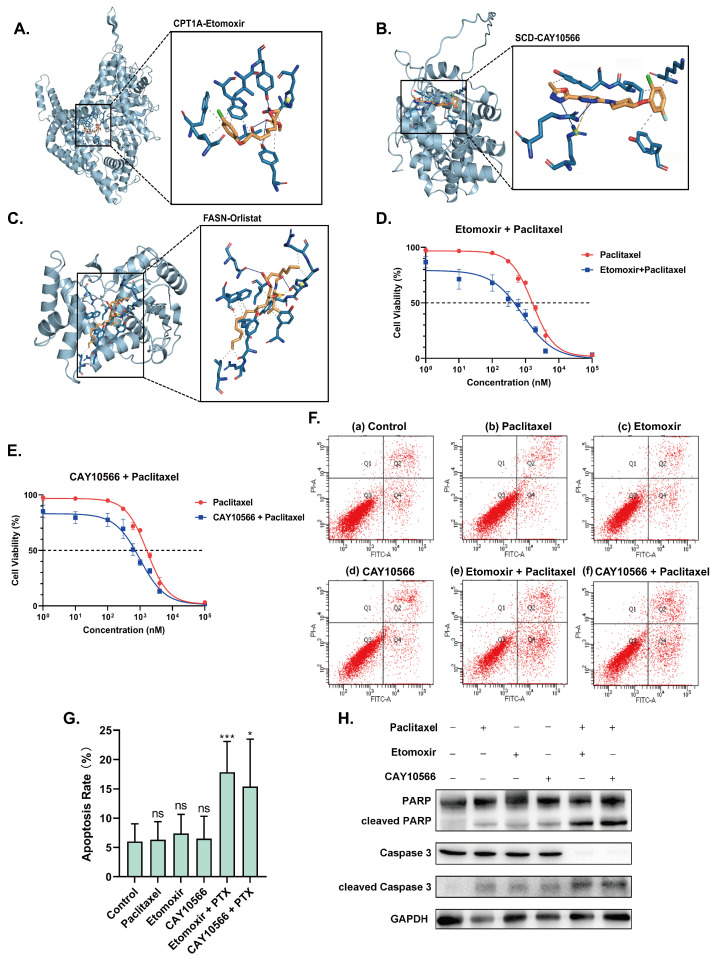
(**A**–**C**) Molecular docking results of CPT1A-etomoxir; SCD-CAY10566; FASN-orlistat and detailed molecular structures of the inhibitors. (**D**,**E**) IC50 curves calculated based on CCK-8 results 48 h after combination treatments of etomoxir (0.03 mM), CAY10566 (0.025 mM) and paclitaxel (0, 0.001, 0.01, 0.1, 0.3, 0.6, 1.0, 2.0, 4.0, 100.0 mM) in A2780/PTX cells. (**F**) The flow cytometric detection of apoptosis with annexin V-AbFlour™ 488/PI double-staining for cells: (**a**) the control, (**b**) paclitaxel (0.3 mM), (**c**) etomoxir (0.03 mM), (**d**) CAY10566 (0.025 mM), (**e**) etomoxir (0.03 mM) and paclitaxel (0.3 mM), (**f**) CAY10566 (0.025 mM) and paclitaxel (0.3 mM) for 48 h in A2780/PTX cells. (**G**) Graph showing apoptosis rates of different treatments with all data presented as mean ± standard deviation. (**H**) Western blot results of apoptosis-related proteins after 72 h of treatments of inhibitors only or combined with paclitaxel. All experiments were performed in triplicate. * represents *p* < 0.05; *** represents *p* < 0.001; ns represents not significant.

Specific functioning sites were located via molecular docking. Affinity is a crucial factor when judging the stability of molecular docking; a more negative binding energy indicates higher affinity. We selected inhibitors that showed the benefit of reducing the activities of the three lipid metabolism-related targets. Autodock vina 1.1.2 and PYMOL were applied to perform virtual docking between CPT1A, SCD, FASN and the corresponding inhibitors. Binding energy < −4 kcal/mol was considered stably combined; all three targets and corresponding inhibitors met the standard. The binding energy for docking between etomoxir and CPT1A was found to be −6.9 kcal/mol (Figure 7A), the docking binding energy between CAY10566 and SCD was −8.2 kcal/mol (Figure 7B), and the binding energy of the docking between orlistat and FASN was −7.7 kcal/mol (Figure 7C). Therefore, we proposed a targeted paclitaxel–inhibitor combination treatment plan for the poor prognosis caused by CPT1A, SCD and FASN, which provides more options for the individualized treatment of patients.

To validate the therapeutical effects of the three targets, etomoxir, CAY10566 and orlistat were applied at concentrations allowing a surviving rate greater than 90% in A2780/PTX cells; inhibitors were then applied combined with paclitaxel. The CCK-8 results showed that the etomoxir+paclitaxel group and the CAY10566+paclitaxel group had the same trend in relation to siRNA knockdown results, but the orlistat+paclitaxel group did not show significance in resensitizing A2780/PTX. The IC50 values of the etomoxir+paclitaxel group and the CAY10566+paclitaxel group were reduced from 1589.5 ± 62.5 nM to 817.4 ± 79.7 nM and 990.1 ± 81.89 nM, respectively (Figure 7D,E).

Flow cytometry analysis was conducted to indicate the apoptosis rates under different treatments. It was observed that the apoptosis rates of applying only paclitaxel, etomoxir and CAY10566 did not show significance compared to those of the control group; however, when combining etomoxir and CAY10566 with paclitaxel, the apoptosis rates were elevated by approximately 10% and 15% (Figure 7F,G). Western blot results of apoptosis-related proteins showed that the expression of cleaved poly ADP-ribose polymerase (cleaved PARP) and cleaved Caspase 3 increased when combining target inhibitors and paclitaxel, which further validated the results of flow cytometry analysis (Figure 7H). Overall, the pharmacological blockade of CPT1A and SCD apparently restored the sensitivity to paclitaxel in the A2780/PTX cell line.

## 3. Discussion

Currently, increasing evidence supports the perspective that lipid metabolism-related enzymes participate in paclitaxel resistance occurrence in EOC, and molecular mechanisms are better understood [36,37]. Acquired paclitaxel resistance in ovarian cancer is considered the result of the synergistic effects of P-glycoprotein and multiple basic biological processes. After integrating transcriptomic data, only *ABCB1* can satisfy the criteria of significant up-regulation in all seven paclitaxel-resistant cell lines. However, the development of P-glycoprotein as a therapeutic target has been unsuccessful [38]; hence, there is an urgent demand for new clinical biomarkers for predicting paclitaxel resistance occurrence and exploration of novel therapeutic strategies.

Given the subjectivity of drug resistance and cancer recurrence evaluations, the application of clinical data to explore the issue of drug resistance is extremely valuable yet difficult, and more refined follow-up records and more objective drug resistance evaluations are required. The Darwinian selection model is based on cancer stem cell theory, which describes a small population of cancer cells that are intrinsically more tolerant to various anticancer agents, is enriched upon drug treatment, which will be progressively mainstreamed in subsequent chemotherapy [39]. The laboratory establishment of paclitaxel-resistant cell lines suits this model. In studies related to drug resistance, exploration at the level of cell lines is easier to conduct; it can assess the degree of drug resistance according to IC50. Meanwhile, transcriptomic data are exclusively obtained from drug-resistant cells; therefore, we chose to use multi-center cell line transcriptomic data as the main research target of our study, instead of low-quality drug resistance-related clinical data.

We applied the original bioinformatic screening process to explore multi-center transcriptomic data, and established a gene set related to acquired paclitaxel resistance and lipid metabolism in ovarian cancer. These genes allow cancer cells the obtention of higher tolerance for abnormalities at a transcriptional level. A2780 and A2780/PTX in different datasets (GSE73935 and GSE159791) can have differences in gene expression; the genetic drift and heterogeneity of the seven pairs of sensitive and resistant cell lines can be magnified. This heterogeneity is due to the differences in methods of establishing resistant cell lines and clinical therapies; therefore, the transcriptomic data obtained from different resistant cell lines and paclitaxel-resistant ovarian cancer patients vary. We selected genes that are up-regulated in most resistant cell lines, and noticed *CPT1A* and *ATP1B1* up-regulated in four cell lines out of seven. The expression of *CPT1A* and *ATP1B1* in pathological tissues was in positive correlation; however, their effects on survival were opposite. We noticed that a higher *CPT1A* expression correlates with a shorter survival period, but a higher *ATP1B1* expression correlates with a prolonged survival time. They play central roles in a wide range of cellular processes, including cell proliferation, survival, migration and chemoresponsiveness. *CPT1A* was found to be highly expressed in ovarian cancer, and its over-expression is linked to a poor survival in ovarian cancer patients [40]. ATP1B1 is the key subunit of Na${}^{+}$/K${}^{+}$ ATPase, playing an important role in the maintenance of adhesion among cancer cells via modulation of tight junctions [41,42,43,44]. Even though over-expression of both *CPT1A* and *ATP1B1* enhances the paclitaxel resistance of ovarian cancer cells, a higher *CPT1A* expression level allows cancer cells the obtention of stronger metastasis ability, while a high *ATP1B1* expression level inhibits metastasis. This difference between CPT1A and ATP1B1 leads to the contradictory effect on survival time. CPT1A is an appealing druggable target for cancer therapies since it is essential for the survival, proliferation, and drug resistance of cancer cells. Therefore, we choose CPT1A as our subsequent research target. Considering CPT1A is the key enzyme in lipid metabolism pathway, we used bio-informatic methods to explore clinical pathological transcriptional profiles, and intersected the gene set related to acquired paclitaxel resistance with the gene set related to lipid metabolism. This novel bio-informatic method confirmed the most suitable cell line for biological validation and application of inhibitors combining paclitaxel. Our research located *CPT1A* as the center gene, and *ACLY*, *ACACA*, *FASN*, *CPT2* and *SCD* as synergistic genes. These enzymes are located in the key joints of lipid metabolism pathways, of which we selected FASN as the up-stream substrate providing the enzyme of CPT1A and SCD as the competitive enzyme sharing the same substrates with CPT1A.

Metabolic reprogramming of a fundamental biological process has notable meaning in the occurrence of paclitaxel resistance in ovarian cancer, and associated key enzymes are important potential aiming targets. More research revealed that lipid metabolism reprogramming was a critical factor of cancer multidrug resistance (MDR), tumorigenesis, and metastasis [45,46,47]. A higher expression of *CPT1A* increases the regular OXPHOS in mitochondria. As a member of the carnitine palmitoyltransferase system, CPT1A is responsible for transporting long-chain acly-CoAs into mitochondria from the cytoplasm, which is the rate-limiting step in β-oxidation [48,49]; therefore, over-expression of *CPT1A* leads to β-oxidation up-regulation [50]. Fatty acid involves the construction of cell structures and forms signaling factors such as a steroid hormone after a series of processing. FASN is the core lipogenic enzyme under a physiological condition, catalyzing the de novo synthesis of fatty acid via consuming acetyl-CoA and malonyl-CoA as materials [51,52]. The changes in FASN activity are thought to be the stress response of cancer cells reacting to changes in cellular micro-environments. It has been reported that hypoxia-inducible factor-1α (HIF-1α) could activate its down-stream target genes and signaling pathways including *FASN* and lipogenesis. In cancer cells, the increased genesis of membranes demands adequate lipids including phospholipids and cholesterol. This requirement is met by increased de novo FA biosynthesis, which has been observed in ovarian cancer [53]. Since the process is regulated mainly by FASN, the over-expression of *FASN* correlates with cancer proliferation, metastases, poor prognosis, and a higher risk of drug resistance. Stearoyl CoA desaturase-1 (SCD1) is an enzyme found in the endoplasmic reticulum (ER) that plays a crucial role in the de novo synthesis of fatty acids. SCD1 catalyzes the conversion of saturated fatty acids (SFAs) into MUFAs such as palmitoleic acid and oleic acid (nonessential fatty acid) [54]. Most MUFAs produced by SCD1 are considered to be materials for phospholipids biosynthesis for continuous membrane biogenesis of dividing cancer cells [55]. SCD1-mediated SFA/MUFA homeostasis is important for cancer risk assessment; the conversion between SFA and MUFA is also closely related to cancer prognosis. The dynamics between SFAs and MUFA regulated by SCD impact ovarian cancer cell survival and tumor progression. Zhao et al. [56] leveraged transcriptomics, lipidomics, and single-cell stimulated Raman scattering microscopy to show that increased levels of lipid unsaturation promoted by SCD protected cancer cells against endoplasmic reticulum stress-induced apoptosis. Over-expression of *SCD* or the exogenous addition of palmitoleic acid can increase the chances of cell survival. SCD also stimulates the Wnt signaling pathway and YAP activation in support of tumorigenesis and facilitates metabolic reprogramming in cancer mediated by the regulation of AKT, AMPK, and NF-κB via MUFAs. Roongta et al. revealed that the expression of *SCD* was up-regulated in ovarian cancer tissues and stem cells [57]. The inhibition of *SCD* expression can induce cancer cell apoptosis: when SCD inhibitors were applied to treat the primary ovarian cancer stem cells, the stemness markers were down-regulated. The accumulation of de novo synthesized SFA in non-fat cells can cause lipotoxicity and induce apoptosis. In *SCD1* overexpressed tumor cells, there is a continuous transformation of SFA to MUFA to avoid lipid toxicity caused by excessive accumulation of SFA. In prostate cancer, SCD1 inhibitor BZ36 can block the conversion from SFA to MUFA, leading to down-regulation of cancer cell viability [39]. Similar effects were observed when applying another SCD1 inhibitor CVT-11127 to lung cancer cells [58].

Evidence also indicates that metabolic phenotypes evolve as cancer progresses, with new dependencies emerging in the context of therapy resistance. Metabolic activation of FAO causes the uncoupling of mitochondrial OXPHOS and facilitates chemoresistance. The decrease in fatty acid de novo synthesis leads to reduced levels of malonyl coenzyme A, which is the metathesis inhibitor of CPT1. Therefore, β-oxidation was activated accompanied by a rise in the *CPT1* expression level. Cancer cells have increased energy production to counteract the toxic effects of chemotherapeutic drugs, resulting in drug resistance [11]. It has been reported that targeting CPT1A could be a beneficial regimen to improve the therapeutic effects of radiotherapy in nasopharyngeal carcinoma (NPC) patients [59]. Huang et al. indicated that CPT1A played a vital role in platinum resistance occurrence in high-grade serous ovarian cancer via RNA-seq, whole-genome sequencing and proteomic profiling comprehensive and reproducible analyses of intra-patient cell line pairs derived from three HGSOC patients before and after acquiring platinum resistance, extending prior work showing that a reduced *CPT1A* expression is associated with platinum sensitivity in a collagen type XI alpha 1 (COL11A1)-dependent in vitro model of platinum resistance [29,60,61]. Since it is widely agreed that platinum resistance often correlates with paclitaxel resistance due to the cross-resistance effect, we aimed to explore the role of CPT1A in the occurrence of paclitaxel resistance in ovarian cancer. The FASN-mediated drug resistance appears to occur due to a decrease in drug-induced apoptosis from an overproduction of palmitic acid by FASN. In addition, FASN is recognized as a therapeutic and chemosensitization target in ovarian cancer tissue, cell lines, and primary cell cultures [62]. Papaevangelou et al. indicated the inhibition of FASN in cisplatin-resistant ovarian cancer cells led to apoptosis when combining orlistat and cisplatin [63]. However, neither CPT1A nor FASN have been reported to correlate with paclitaxel resistant in ovarian cancer, and to the best of our knowledge, there is no research about SCD participating in drug resistance in ovarian cancer, yet it regulates sorafenib resistance via the modulation of ER stress-induced differentiation in hepatocellular carcinomas (HCCs) [64] Our systematic research of the three targets associated with paclitaxel resistance in ovarian cancer is important in understanding the detailed mechanisms of paclitaxel resistance occurrence. We observed the protein over-expression of CPT1A, SCD and FASN in the paclitaxel-resistant cell line compared to that of the paclitaxel-sensitive cell line. The knockdown of *CPT1A*, *SCD* and *FASN* restored the sensitivity of paclitaxel in A2780/PTX cells. In paclitaxel-resistant cells, cellular metabolism alteration supports proliferation when facing cellular micro-environmental stress after chemotherapy application.

Metabolic phenotypes and dependencies evolve as cancer progresses from preneoplastic lesions to localized, clinically apparent malignancies to metastatic cancer. The development of ovarian cancer progression and metastatic dissemination requires an adequate energy supply, which correlates to cancer phenotype requirements, meanwhile facilitated by the interactions between cancer cells and the micro-environment. Ovarian tumor cells depend on these adipocyte-derived fatty acids to support rapid growth and continued peritoneal dissemination [65]. The uptake of fatty acids from surrounding adipocytes promoted FAO in cancer cells [66,67]. In particular, ovarian cancer growths from adipocyte-rich environments is more likely to be FAO-addicted forms of cancers. Abnormal lipid metabolism has been identified as an emerging feature of cancer cells because it leads to dysregulations in the expression of genes, proteins, and signaling transduction pathways that directly or indirectly participate in cancer metastasis. Fatty acid oxidation supports colonization of lymph nodes by melanoma [68]. Triple-negative breast cancers rely on fatty acid oxidation to maintain aberrant Src activity, which promotes metastasis [23]. Lipid-rich microenvironment may cause epigenetic silencing miR-33b, which negatively modulates ovarian cancer peritoneal metastases by suppressing TAK1/FASN/CPT1A/NF-κB signaling [69]. According to Xiong et al., CPT1A regulates the expression of vascular endothelial growth factor (VEGF), which modulates vascular permeability and promotes cancer cell migration, leading to further deterioration [28]. FASN plays a critical role in the peritoneal metastasis of ovarian cancer. Targeting de novo lipogenesis may have a therapeutic potential for advanced ovarian cancer [70]. Moreover, the up-regulation of *FASN* expression can further stimulate the processes of endothelial cell recruitment and induction of the vasculature of cancer cells, which promotes cell growth and correlates with poor prognosis [71,72]. Studies of SCD correlating with metastases are very rare and require extensive investigation. In this study, we explored whether the inhibition of *CPT1A*, *SCD* and *FASN* could influence other biological processes apart from cell growth. Results of Transwell migration assay indicated that the inhibition of *CPT1A*, *SCD* and *FASN* led to significant decrease in the metastasis ability of paclitaxel-resistant cells in absence of chemotherapy. The co-up-regulation of *CPT1A*, *SCD* and *FASN* indicated that paclitaxel resistance occurrence is a complicated event which involves multiple metabolic processes, including promoted lipid de novo biosynthesis that meets the need of energy supply and materials required for cancer metastases and invasion.

Several metabolic inhibitors designed to target lipid metabolism pathways have advanced into preclinical trials and clinical trials; CPT1A, SCD and FASN can be promising therapeutic targets. The inhibitors of CPT1A, etomoxir and perhexiline, were applied to enhance carboplatin sensitivity, which reduced tumor growth in vivo significantly when combined with carboplatin [29]. Tesfay et al. [73] reported that *SCD1* was over-expressed in ovarian cancer, that the pharmacological blockade of SCD1 using CAY10566 induces apoptosis, and that, in addition, the inhibition of SCD1 can also enhance the sensitivity to ferroptosis inducers in ovarian cancer cell lines. Bauerschlag et al. [62] reported that a continuous application of FASN inhibitor cerulenin combined with cisplatin can prominently reduce the IC50 value to cisplatin in cisplatin-resistant ovarian cancer cells, and that cisplatin-resistant cells show a decreased intake of ${}^{18}$F-fluoromethylcholine (${}^{18}$F-FCH), suggesting that metabolic imaging may be able to guide treatments. Our study applied etomoxir to block CPT1A, CAY10566 to block SCD, and orlistat to block FASN. When conducting treatments combining inhibitors with paclitaxel, both etomoxir and CAY10566 restored the sensitivity to paclitaxel of resistant ovarian cancer cells. However, when applying the combination of orlistat and paclitaxel, the resistant ovarian cancer cell line did not resensitize to paclitaxel, although previous research indicated that orlistat can reverse the resistance to cisplatin in ovarian cancer. Our failure may be the result of differences in mechanisms of drug resistance, details of which require further research. CAY10566 was observed to inhibit oleic acid synthesis in the HCT116 colon cancer in vitro assay, inducing PARP degradation, inhibiting cell growth and promoting apoptosis. We also observed the expression levels of activated apoptosis-related proteins, cleaved PARP and cleaved Caspase 3, increased when combining target inhibitors and paclitaxel. These results indicate that inhibitors interfered with the dysfunctional metabolic processes required for cancer cells to survive in a stressful environment, causing paclitaxel-resistant ovarian cancer cells to become vulnerable to chemotherapy, finally leading to apoptosis.

Even though we developed novel bio-informatic methods for potential paclitaxel resistance biomarker screening, there are still several limitations in our research. We did not provide direct in vivo experiment results to uncover the detailed mechanisms of *CPT1A*, *SCD* and *FASN* over-expression stimulating cancer cell proliferation and metastases. Therefore, it is necessary to explore a combined intervention using a pharmacological inhibition of FAO, FA-enriched diet and paclitaxel in nude mice. Furthermore, we lack first-hand clinical data. The correlation between the expression of *CPT1A*, *SCD* and *FASN* and clinicopathological features requires further exploration.

## 4. Materials and Methods

### 4.1. Data Collection

To reveal key genes involved in the regulation of acquired paclitaxel resistance in ovarian cancer, transcriptomic data were retrieved from the GEO database (https://www.ncbi.nlm.nih.gov/geo/, accessed on 26 September 2022). Select datasets in the GEO database using Ovarian cancer AND Paclitaxel as keywords were obtained. The inclusion criteria of GEO datasets were as follows: Guaranteed stability of paclitaxel resistance in ovarian cancer cell lines while providing data of corresponding sensitive ovarian cancer cell lines as control. Datasets without quality control of the expression profiles and studies in which the stability of paclitaxel resistance could not be guaranteed due to transient paclitaxel treatment in cell lines were excluded. We obtained five GEO datasets that met the criteria (GSE73935, GSE172016, GSE60335, GSE26465, GSE159791), and analyzed the sensitive groups and resistant groups. Notably, due to significant cross-resistance between adriamycin and paclitaxel [34], we also considered 4 adriamycin-resistant cell lines in GSE26465 as multidrug-resistant groups.

**Figure 8:**
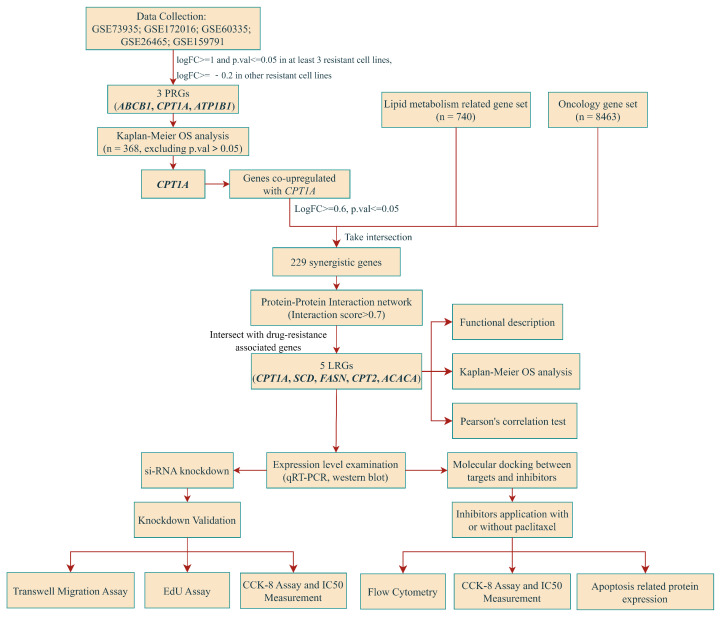
Workflow of this study.

In total, 368 pathological tissue expression profiles, phenotype and survival data of patients were obtained from the GDC TCGA Ovarian Cancer (OV) cohort of UCSC Xena (https://xenabrowser.net/data-pages/, accessed on 12 January 2023, gene expression RNAseq-HTSeq-Counts (version 07-20-2019), phenotype-Phenotype (version 08-07-2019), phenotype-survival data (version 07-20-2019)). Age, FIGO grading, tumour histological grading and transcriptome data of pathology samples were obtained from 368 patients, and patient data were selected according to the following criteria: (i) patients without survival data were excluded, (ii) patients without records of age at first diagnosis were excluded, (iii) patients without records of FIGO grading or histological grading of tumors were excluded, (iv) patients who did not provide or provided more than one pathology sample were excluded, and (v) patients without transcriptome data from pathology samples were excluded (Figure 8).

### 4.2. Data Process and Candidate Genes Screening

Analysis was applied for 7 paclitaxel-resistant cell lines using the “limma” R package, expression profiles with significant batch effects were standardized using “normalizeBetweenArrays()” in the “limma” R package. Genes which up-regulate significantly in at least 3 paclitaxel-resistant cell lines (logFC ≥ 1 and *p*.val ≤ 0.05) and meet the logFC ≥ −0.2 criterion in other paclitaxel-resistant cell lines were considered as candidate genes. Candidate genes were then ranked according to the frequencies of significant up-regulation in resistant cell lines. Candidate genes with the highest rankings were defined as PRGs. Paired samples *t*-test was applied to confirm the significance of a higher expression of PRGs in all 7 paclitaxel-resistant ovarian cancer cell lines compared to corresponding sensitive cell lines.

### 4.3. Overall Survival Analysis and Screening Genes with Synergistic Effect

The transcriptomic data of pathological samples from GDC TCGA Ovarian Cancer (OV) was applied to verify the expression correlation between *CPT1A* and *ATP1B1* and univariate regression analysis was performed. The Kaplan–Meier overall survival analysis of PRGs was performed using data from GDC TCGA Ovarian Cancer (OV). *CPT1A* was found to be a survival-related gene using “survival” and “survminer” R packages. Patients were divided into two groups based on the median expression level of *CPT1A*.

Limma analysis was applied to screen up-regulated genes (logFC ≥ 0.6 and *p*.val ≤ 0.05) in the higher *CPT1A* expression group (Table S1). Considering the special status of CPT1A in lipid metabolism, we established 2 gene sets: the lipid metabolism-related gene set and the oncological gene set. The results of the gene sets are provided in Tables S2 and S3. Data sources of gene sets are PathCards (https://pathcards.genecards.org/, accessed on 12 January 2023) and GeneCards (https://www.genecards.org/, accessed on 12 January 2023).

Therefore, the synergistic gene set of *CPT1A* is defined as genes involved in the lipid metabolism-related gene set, the oncological gene set and the co-upregulated genes of *CPT1A* at the same time (Table S4). Venn diagrams were constructed using http://www.ehbio.com/test/venn/#/ (accessed on 23 February 2023) (Supplementary Material S1).

### 4.4. Further Identification of CPT1A Synergistic Genes via Protein–Protein Interaction Networks and Survival Analysis

The protein–protein interaction (PPI) network of the synergistic genes of *CPT1A* was constructed using the Search Tool for the Retrieval of Interaction Genes (STRING) database (https://cn.string-db.org/, accessed on 16 March 2023). Select highly confidential nodes (interaction scores > 0.7) connected to CPT1A in the network as core synergistic genes may influence the CPT1A function in a synergistic manner. Furthermore, we took the intersection of core synergistic genes with genes mentioned in the review drafted by Yang et al. [35], who correlated lipid metabolism reprogramming and cancer drug resistance, and obtained lipid metabolism-related paclitaxel resistance-associated genes (LRGs). We then applied the Kaplan–Meier plotter (https://kmplot.com/analysis/, accessed on 16 March 2023) to perform overall survival analysis of the LRGs chosen for further biological experiments.

### 4.5. Cell Culture and Reagents

Human epithelial ovarian cancer cell line A2780 was purchased from Nanjing KeyGen Biotech Co., Ltd. Cells were cultured in DMEM (PM150210, Procell, Wuhan, China) containing 10% fetal bovine serum (164210-50, Procell) and a penicillin–streptomycin solution (PB180120, Procell) in an incubator at 37 °C and 5% CO${}_{2}$.

### 4.6. Establishment of Paclitaxel-Resistant Cell Lines

A2780/PTX derived from the A2780 parental cell line were developed by growing A2780 cells instep-wise increases in paclitaxel doses following general instructions, partly according to Coley et al. [74]. A2780 cells were seeded to a tissue culture flask and allowed adherence for 24 h. The next day, when the culture was about 50% confluent, paclitaxel was added to 0.1 nM of the final concentration in the medium and cells were grown until confluency became about 90% (meanwhile, the medium with paclitaxel was changed every 2–3 days). For the next selection process, a paclitaxel dose (0.5 nM) was added in the medium. This procedure was continued, generally followed by the pattern of doubling the paclitaxel concentration after cells reached the stable tolerance to the former dose. Once the cells appeared not to tolerate the consecutive paclitaxel dose, they were allowed growth in a drug-free medium for recovery, until growth appeared healthy and confluency reached about 90%. This selection process was repeated for several times using a double concentration of paclitaxel in the previous step until all cells could survive at the paclitaxel concentration of 500 nM. The surviving cells were able to maintain the paclitaxel-resistant phenotype in the absence of the selection pressure and were named A2780/PTX. The total process of establishing resistant cells took about 12 months.

### 4.7. RNA Extraction and Quantitative Real-Time PCR

Total mRNA was extracted from cells using RNAiso Plus (D9108B, Takara, Beijing, China) according to the manufacturer’s protocol. We synthesized cDNA by using a PrimeScript™ RT reagent Kit with gDNA Eraser (Perfect Real Time) (RR047A, Takara) according to the manufacturer’s protocol. Quantitative real-time PCR was performed using TB Green^®^ *Premix Ex Taq*™ II (Tli RNaseH Plus) (RR820A, Takara) and a LightCycler 480 version 1.5.1.62 (Roche, Basel, Switzerland). The primer sequences of the target genes are listed in Supplementary Material S2. Data were calculated using the 2${}^{-\Delta \Delta Ct}$ method.

### 4.8. Western Blot

All proteins were collected at 4 °C. BCA assay was performed to quantify protein concentration. A total of 5 μL of proteins was isolated via SDS-PAGE and transferred to 0.45 μm and 0.22 μm polyvinylidene fluoride (PVDF) membranes (BS-PVDF-45, BS-PVDF-22, Biosharp, Hefei, China). Then, the membranes were blocked with 5% non-fat milk for 1 h at room temperature and incubated with primary antibodies at 4 °C overnight, followed by incubation with HRP secondary antibodies for 1 h at room temperature. Primary antibodies included anti-SCD (1:2000; 28678-1-AP, Proteintech, Wuhan, China), anti-FASN (1:2000; 10624-2-AP, Proteintech), anti-CPT1A (1:5000; 15184-1-AP, Proteintech), anti-PARP (1:1000, 13371-1-AP, Proteintech), anti-Caspase 3 (1:1000, 19677-1-AP, Proteintech), and anti-GAPDH (1:5000; 60004-1-Ig, Proteintech) was utilized for normalization.

### 4.9. Transfection of Small Interfering RNA

To down-regulate the expression of *CPT1A*, *SCD* and *FASN*, siRNA against CPT1A (si-CPT1A, GenePharma, Shanghai, China), SCD (si-SCD, GenePharma) and FASN (si-FASN, GenePharma) were transfected into A2780/PTX cells (Supplementary Material S2). Lipofectamine 2000 (Invitrogen, Carlsbad, CA, USA) was applied according to the manufacturer’s instructions during the transfection of si-CPT1A, si-SCD, si-FASN and siRNA of the negative control (si-NC, GenePharma).

### 4.10. Obtention and Validation of Inhibitors of CPT1A, FASN and SCD

We used Autodock vina 1.1.2 and PYMOL to perform molecular docking of CPT1A, SCD, and FASN with their inhibitors etomoxir, CAY10566, and orlistat, respectively. Structure profiles of protein and inhibitors were obtained from the AlphaFold Protein Structure Database (https://www.alphafold.ebi.ac.uk/, accessed on 1 November 2023) and the DrugBank database (https://go.drugbank.com/, accessed on 1 November 2023). Binding energy < −4 kcal/mol was considered a stable combination when assessing the stability of molecular docking.

### 4.11. Cell Viability Assay

A CCK-8 kit (GK10001, GlpBio, Montclair, CA, USA) was used for the cell viability test. A total of 5 × 10${}^{3}$ cells were seeded in a 96-well plate after siRNA knockdown or treated using etomoxir (GC16736, GlpBio), CAY10566 (GC18558, GlpBio) and orlistat (O4139, Merck, Darmstadt, Germany). Different concentrations of paclitaxel were added in corresponding wells after 24 h. At 24 h, 48 h and 72 h, 10 μL of the CCK-8 reagent was added into each well and incubated at 37 °C for 2 h. A microplate reader (Spark 10M, Tecan, Männedorf, Switzerland) was used to measure the absorbance at a 450 nm wavelength.

The EdU assay was performed according to manufacturer’s protocol (CX002, Epizyme, Shanghai, China). The results were processed using ImageJ software (version 1.46r, Bethesda, MD, USA).

### 4.12. Cell Colony Formation Assay

A2780/PTX cells were transfected with si-CPT1A, si-FASN, and si-SCD for 48 h, then planted in 6-well plates at a density of 400 cells per well. Cells were treated using paclitaxel at concentrations of 20 nM, 40 nM and 80 nM after 7 days of incubation. After 14 days, the cells were fixated with 4% paraformaldehyde at room temperature for 30 min and stained with 0.25% crystal violet at room temperature for 30 min. Finally, the formed colonies were imaged and counted using ImageJ software. A2780/PTX cells were also transfected with si-NC for comparison.

### 4.13. Transwell Cell Migration Assay

Transwell chambers (#3422, Costar, Corning, NY, USA) were used to detect the migration ability of paclitaxel-resistant ovarian cancer cells. A total of 1 × 10${}^{5}$ A2780/PTX cells transfected with si-CPT1A, si-SCD, si-FASN and si-NC were seeded in a 200 μL RPMI-1640 serum-free medium in Transwell chambers; another 600 μL RPMI-1640 medium with 10%FBS was added as a chemoattractant in a corresponding 24-well plate. After incubation for 48 h, chambers were washed using PBS and cells were fixated using 4% paraformaldehyde for 15 min at room temperature. Chambers were dyed with 0.25% crystal violet solution for 20 min after cleaning paraformaldehyde. Photomicrographs of the Transwell chambers were taken using an inverted microscope (ECLIPSE Ts2, Nikon, Tokyo, Japan) and the number of migrated cells was counted using ImageJ software.

### 4.14. Flow Cytometry

A2780/Taxol cells were pretreated separately using etomoxir and CAY10566 with or without paclitaxel for 48 h. A total of 1 × 10${}^{6}$ cells at the logarithmic phase of growth were harvested according to manufacturer’s protocol: rinsed twice with PBS, each sample resuspended using a 100 μL 1 × Annexin V binding buffer, 5 μL of Annexin V-AbFlour™ 488 and 2 μL of propidium iodide (PI) (KTA0002, Abbkine, Wuhan, China) added and incubated on ice in the dark for 15 min. Apoptosis rates were detected in a FACS Celesta flow cytometer (Becton Dickinson and Company, Franklin Lakes, NJ, USA); 1 × 10${}^{4}$ events per sample were analyzed using MODFIT software (Becton Dickinson and Company).

### 4.15. Statistical Analysis

Data were analyzed using R 4.0.5 (Supplementary Material S3) and GraphPad Prism 9.4.0 statistical software (GraphPad Software Inc., La Jolla, CA, USA). All experiments were performed in triplicate, and data are shown as the means ± standard deviation (means ± SD). The differences between two groups were tested by paired samples *t*-tests. One-way ANOVA was used for analysis between three or more groups. *p* values smaller than 0.05 were considered statistically significant (ns represents not significant; * represents *p* < 0.05; ** represents *p* < 0.01; *** represents *p* < 0.001; **** represents *p* < 0.0001).

## 5. Conclusions

Through a series of bio-informatic analyses and in vitro experiments, the key enzymes over-activated in metabolic reprogramming during the progression of EOC paclitaxel resistance occurrence were extensively investigated. Therefore, it was validated that paclitaxel-resistant EOC patients have higher *CPT1A*, *SCD* and *FASN* expression levels. The pharmacological inhibition of the three targets significantly down-regulated the proliferation and metastasis ability of paclitaxel-resistant EOC cells. Our results highlight the importance of combination strategies of pharmacological inhibition of FAO combined with paclitaxel as metabolic interventions to target paclitaxel-resistant ovarian cancer.

## Figures and Tables

**Table 1:** The clinical characteristics of the 368 patients.

Disease Characteristics	All (N = 368)
Age at diagnosis	
Median	59
Range	30–87
> 65	114 (31%)
Grade	
G1	1 (0.3%)
G2	39 (10.6%)
G3	319 (86.7%)
G4	1 (0.3%)
GB	2 (0.5%)
GX	6 (1.6%)
FIGO stage	
Stage I	1 (0.3%)
Stage II	21 (5.7%)
Stage III	289 (78.5%)
Stage IV	57 (15.5%)

## Data Availability

Data available on request due to restrictions, e.g., privacy or ethical.

## Supplementary Materials

The following supporting information can be downloaded at: https://www.mdpi.com/article/10.3390/ijms242216503/s1.
