# Supplementary material for: Resensitizing Paclitaxel-Resistant Ovarian Cancer via Targeting Lipid Metabolism Key Enzymes CPT1A, SCD and FASN
Source: Int J Mol Sci. 2023 Nov 19;24(22):16503. doi: 10.3390/ijms242216503 (PMC10671839; doi:10.3390/ijms242216503)
Supplement: Supplementary file 1 [file ijms-24-16503-s001.zip › Supplementary material 2.pdf]

1. qRT-PCR primer sequences

Gene name	Forward primer (5'-3')	Reverse primer (5'-3')
CPT1A	CTGGACAATACCTCGGAGCC	AACGTCACAAAGAACGCTGC
SCD	CTTGCGATATGCTGTGGTGC	CCGGGGGCTAATGTTCTTGT
FASN	CAACTCACGCTCCGGAAA	TGTGGATGCTGTCAAGGG
GADPH	GAAGGTGAAGGTCGGAGTC	GAAGATGGTGATGGGATTTC

2. siRNA sequences

Gene name	Sense (5'-3')	Antisense (5'-3')
si-CPT1A	GGUGGGAGGAAUACAUCUATT	UAGAUGUAUUCCUCCCACCTT
si-SCD	CUACGGCUCUUUCUGAUCATT	UGAUCAGAAAGAGCCGUAGTT
si-FASN	UGAGAAAGGUCGAAUUUGCCA	UGGCAAAUUCGACCUUUCUCA
si-NC	UUCUCCGAACGUGUCACGUTT	ACGUGACACGUUCGGAGAATT
