# Supplementary figures and images for: Resensitizing Paclitaxel-Resistant Ovarian Cancer via Targeting Lipid Metabolism Key Enzymes CPT1A, SCD and FASN
Source: Int J Mol Sci. 2023 Nov 19;24(22):16503. doi: 10.3390/ijms242216503 (PMC10671839; doi:10.3390/ijms242216503)

– Figure 1: Lipid metabolism gene set

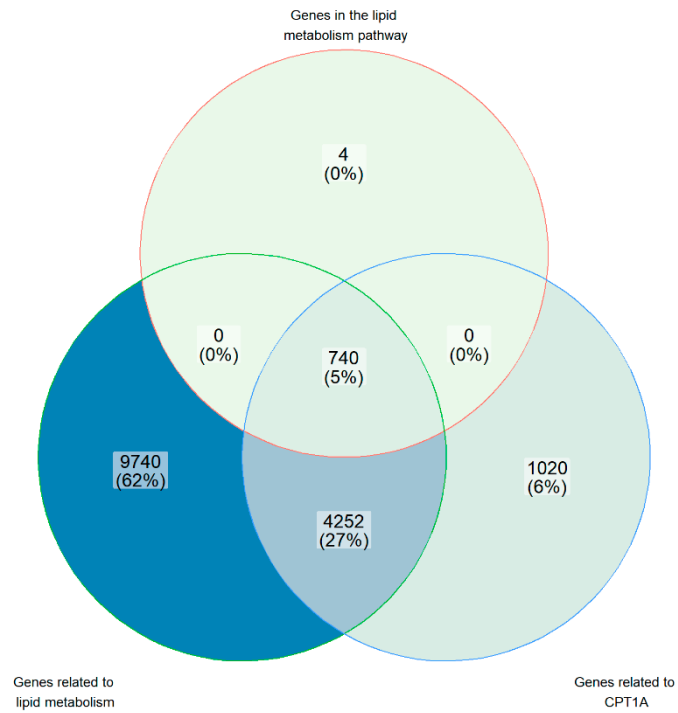

– Figure 2: Oncology gene set

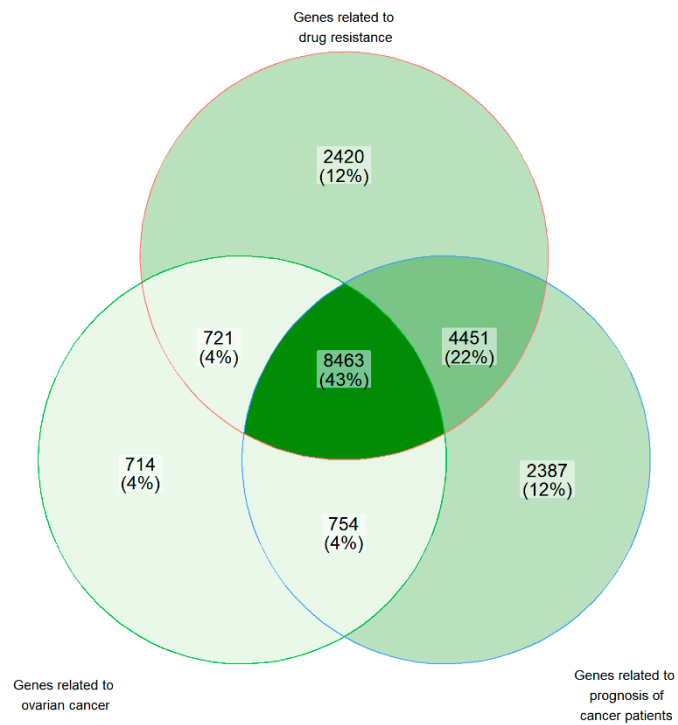

Figure 3: CPT1A synergistic gene set

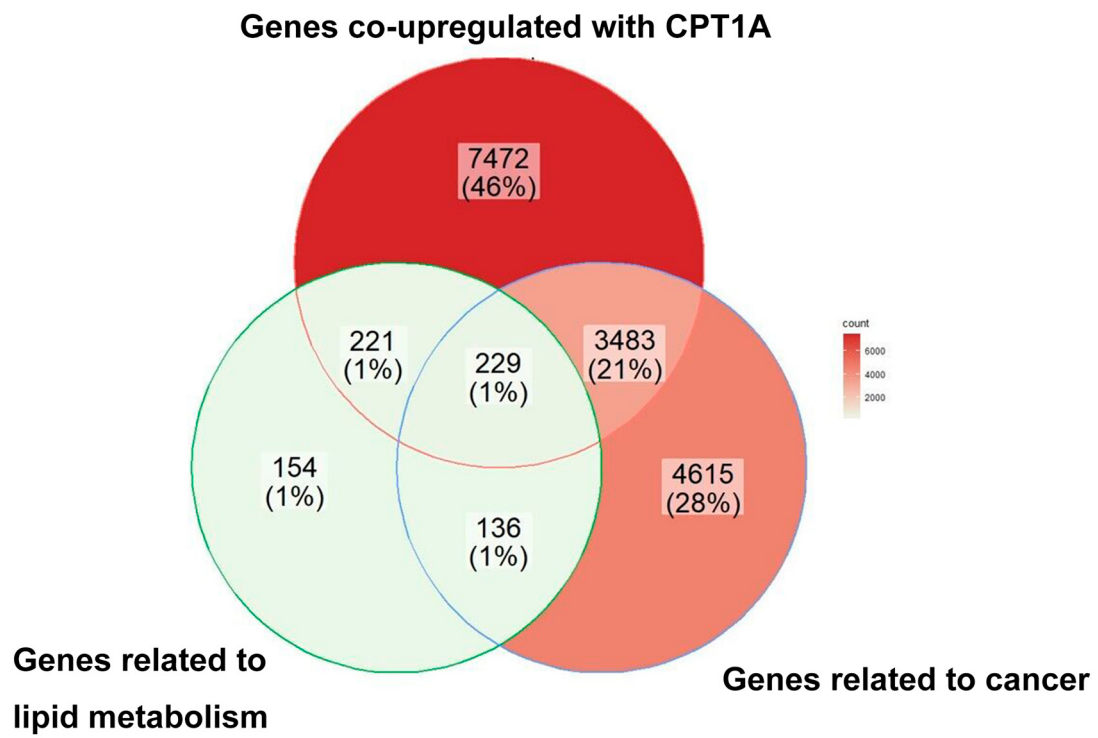

Supplement: Supplementary file 1 [file ijms-24-16503-s001.zip › Supplementary material 1.pdf]
