# Supplementary material for: Resensitizing Paclitaxel-Resistant Ovarian Cancer via Targeting Lipid Metabolism Key Enzymes CPT1A, SCD and FASN
Source: Int J Mol Sci. 2023 Nov 19;24(22):16503. doi: 10.3390/ijms242216503 (PMC10671839; doi:10.3390/ijms242216503)
Supplement: Supplementary file 1 [file Supplementary material 3.pdf › Supplementary material 3.pdf]

1. R packages used in this research:

GEOquery
limma
rio
tidyverse
tidyr
dplyr
R.utils
UpSetR
ggplot2
stats
org.Hs.eg.db
clusterProfiler
data.table
magrittr
survival
survminer
rms
grid
lattice
Formula
Hmisc
clusterProfiler
org.Hs.eg.db
clusterProfiler
DOSE
topGO
pathview
circlize
ggsci
ggplot2
scales
pheatmap
ggrepel
waffle
GO.db
ggVennDiagram
corrgram
corrplot
ggpubr
ggvenn
